# FOXP3 Expression Is Upregulated in CD4^**+**^T Cells in Progressive HIV-1 Infection and Is a Marker of Disease Severity

**DOI:** 10.1371/journal.pone.0011762

**Published:** 2010-07-23

**Authors:** Melinda S. Suchard, Elizabeth Mayne, Victoria A. Green, Sharon Shalekoff, Samantha L. Donninger, Wendy S. Stevens, Clive M. Gray, Caroline T. Tiemessen

**Affiliations:** 1 Haematology and Molecular Medicine, National Health Laboratory Service and University of the Witwatersrand, Johannesburg, South Africa; 2 Microbiology, National Health Laboratory Service and University of the Witwatersrand, Johannesburg, South Africa; 3 Department of Biology and Biochemistry, University of Bath, Bath, United Kingdom; 4 AIDS Virus Research Unit, National Institute for Communicable Disease, Johannesburg, South Africa; New York University, United States of America

## Abstract

**Background:**

Understanding the role of different classes of T cells during HIV infection is critical to determining which responses correlate with protective immunity. To date, it is unclear whether alterations in regulatory T cell (Treg) function are contributory to progression of HIV infection.

**Methodology:**

FOXP3 expression was measured by both qRT-PCR and by flow cytometry in HIV-infected individuals and uninfected controls together with expression of CD25, GITR and CTLA-4. Cultured peripheral blood mononuclear cells were stimulated with anti-CD3 and cell proliferation was assessed by CFSE dilution.

**Principal Findings:**

HIV infected individuals had significantly higher frequencies of CD4^+^FOXP3^+^ T cells (median of 8.11%; range 1.33%–26.27%) than healthy controls (median 3.72%; range 1.3–7.5%; P = 0.002), despite having lower absolute counts of CD4^+^FOXP3^+^ T cells. There was a significant positive correlation between the frequency of CD4^+^FOXP3^+^ T cells and viral load (rho = 0.593 P = 0.003) and a significant negative correlation with CD4 count (rho = −0.423 P = 0.044). 48% of our patients had CD4 counts below 200 cells/µl and these patients showed a marked elevation of FOXP3 percentage (median 10% range 4.07%–26.27%). Assessing the mechanism of increased FOXP3 frequency, we found that the high FOXP3 levels noted in HIV infected individuals dropped rapidly in unstimulated culture conditions but could be restimulated by T cell receptor stimulation. This suggests that the high FOXP3 expression in HIV infected patients is likely due to FOXP3 upregulation by individual CD4^+^ T cells following antigenic or other stimulation.

**Conclusions/Significance:**

FOXP3 expression in the CD4^+^ T cell population is a marker of severity of HIV infection and a potential prognostic marker of disease progression.

## Introduction

Many aspects of HIV pathogenesis are still poorly understood. Despite the CD4 T cell depletion and resulting immunosuppression which are hallmarks of the disease, HIV infected individuals display increased levels of immune activation as evidenced by elevated expression of markers of cell activation such as HLA-DR, CD38 and CD69 [1], [2]. The contributory role of preservation or destruction of regulatory T cells (Tregs), either in number or function, has not been established.

Naturally occurring Tregs are a subset of CD4^+^ T cells expressing the forkhead-winged-helix transcription factor, Forkhead box 3 or FOXP3 [3]. They are responsible for immunoregulation predominantly through cell-cell contact mediated suppression. It is plausible that preferential destruction or inactivation of Tregs by HIV could lead to excessive immune activation [4], [5]. Treg-mediated suppression of HIV specific responses *in vitro* has been shown to be more effective with cells isolated from relatively healthy HIV infected patients compared with later stage AIDS patients, suggesting that Tregs (total or HIV-specific) were depleted or dysfunctional later in HIV disease [6].

On the other hand, preferential preservation of Tregs over other subsets of T cells could lead to suppression of immune responses to viral infections, leading to a high viral load [7], [8]. Various authors have described increased Tregs as a proportion of CD4^+^ T cells in HIV positive patients, particularly in those with low CD4^+^ T cell counts (CD4 counts) [9], [10], [11]. In peripheral blood, Treg levels have been reported to remain elevated years after successful highly active antiretroviral therapy [9], [12], [13]. Certain authors [14] have suggested that with progression from HIV to AIDS the number of circulating CD4^+^CD25hi Treg as a proportion of CD4 T cells increases but their function (which was measured by *FOXP3* mRNA expression) decreases.

Generation of T cells expressing FOXP3 or with suppressor activity has been reported to occur through incomplete activation of CD4^+^ T cells by immature, plasmacytoid or alternatively-activated dendritic cells [15], [16], [17], [18], [19], [20], [21] in HIV-infected individuals as well as in response to Vitamin D [22], all trans retinoic acid [23], [24], [25], [26] or indoleamine deoxygenase (IDO) modulation in antigen presenting cells [20], [27], [28], [29], [30], [31], [32]. One or more of these mechanisms may by responsible for alteration of FOXP3 expression in HIV infected individuals. The end result may be a disproportionate increase in cells with a suppressive or tolerant rather than a proinflammatory phenotype, resulting in an inability of the host to combat pathogens.

There are a number of reasons why different studies may have had contrasting findings. Principally, there is still no validated marker with which to identify human Tregs. The best available to date is forkhead box transcription factor P3, written “*Foxp3*” in animals and “*FOXP3*” in humans. FOXP3 is a key control element in the development and function of CD4^+^ T cells with suppressor function [33], [34], [35], [36].

Use of other markers to identify Tregs have varied from study to study and include CD25, Cytotoxic T lymphocyte associated protein 4 (CTLA-4 or CD152), glucocorticoid-induced tumour necrosis factor receptor (GITR), CD27, OX40 ([37], CD44 [38], CD62L [39], [40], CD39 [41] and decreased expression of CD127 [42], [43], [44]. Tregs are generally thought to be of memory phenotype expressing CD45RO [8], [45], [46], although there have been reports of naïve CD45RA Tregs [47], [48], [49]. None of these markers are exclusive to the Treg population and many are also expressed by activated CD4^+^ T cells.

While Tregs are easily defined in mice by concurrent expression of FOXP3 and high levels of CD25, in humans CD25 cannot be clearly delineated into low and hi expressing subsets due to a continuum of expression. In addition, CD25 expression does not always correlate with FOXP3 expression in humans, particularly in HIV infected individuals [31]. FOXP3 expression can be triggered in FOXP3^−^ CD4^+^ T cells during activation or division which may correlate with, perhaps transient, suppressive potential [50], [51], [52], [53], [54], [55]. There is also however conflicting evidence suggesting that FOXP3 expression in humans may not be confined to cells with regulatory function [44], [56], [57], [58], [59].

We analysed FOXP3 expression, as well as other Treg markers, in South African patients with and without HIV infection. We found a significantly elevated percentage of FOXP3 expressing CD4^+^ T cells in HIV infected patients, particularly in those with lower CD4 counts. We explored the mechanism of FOXP3 upregulation by assessing the ability of cultured cells from HIV positive and negative patients to maintain FOXP3 expression in unstimulated conditions and following T cell receptor stimulation. While many previous studies have isolated CD4^+^CD25^hi^ cells and mixed them with responder cells in a predetermined ratio such as 1∶1 or 1∶10, we avoided this approach as it does not reflect the situation *in vivo* at a physiological ratio of Tregs to responder cells. Additionally the isolation of Tregs by CD25hi sorting potentially leaves FOXP3-expressing Tregs behind in the responder population. We sought to investigate FOXP3 expression after T cell receptor stimulation while maintaining a physiological ratio of Tregs to responder cells. FOXP3 expression dropped rapidly in unstimulated cell culture but was restored by T cell receptor stimulation. This suggests that the high FOXP3 expression in HIV infected patients is likely due to FOXP3 upregulation by individual CD4^+^ T cells following antigenic or other stimulation.

## Materials and Methods

### Clinical Samples

**Figure 1:**
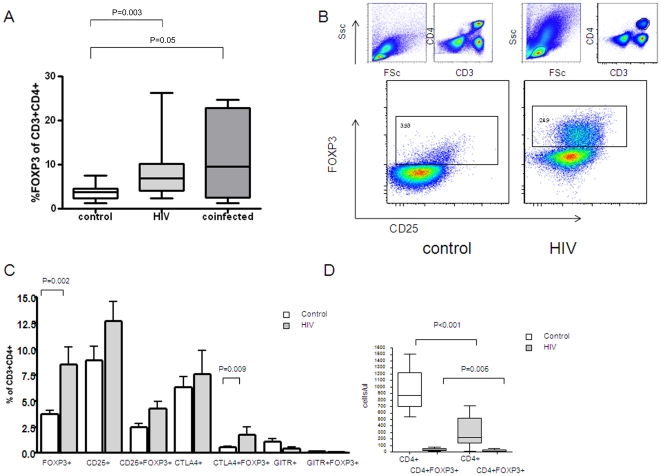
FOXP3 expression at baseline in HIV infected patients. *Panel A*: Subgroup analysis of the HIV and HIV/TB coinfected groups showed no difference in FOXP3 expression as a percentage of CD3^+^CD4^+^ T lymphocytes between the HIV and HIV/TB co-infected groups. *Panel B*: Representative plots of baseline FOXP3 expression in CD3^+^CD4^+^ lymphocytes plotted against CD25 for a control and an HIV positive sample. *Panel C*: Expression of FOXP3, CD25, CTLA-4 and GITR as a percentage of the CD3^+^CD4^+^ population. *Panel D*: Absolute numbers of total and FOXP3 expressing CD4^+^ T cells at baseline.

In this cross-sectional study, twenty-seven HIV infected patients were recruited from the antiretroviral clinics of the Charlotte Maxeke Johannesburg Academic hospital and health care centres in Alexandra township, Johannesburg. Ten of the patients had active Tuberculosis as diagnosed on the basis of symptoms and sputum microscopy. As the HIV-infected patients with and without active Tuberculosis did not differ from each other with regards to CD4 count or FOXP3 expression (Figure 1A), they were grouped together for further analysis. Patients had access to the national antiretroviral programme but had not yet commenced antiretroviral therapy or received more than four days of TB treatment at study enrolment.

Twenty-two healthy controls were recruited from amongst health-care workers of the institutions named above as well as blood donors from the South African National Blood Transfusion Service. Controls and HIV infected subjects were similar with respect to gender and age. All subjects gave written informed consent for the study and ethics approval was granted by the University of the Witwatersrand Human Medical Ethics Committee (R14/49). The day of sample collection was regarded as Day 0.

### HIV tests and haematological parameters

The HIV status of participants was confirmed using HIV rapid testing (*Determine HIV-1/2*, Abbott Laboratories, Abbot Park, Il 60064,USA). CD4 counting was performed using the PanLeucogating method [60]. Viral loads were performed using the COBAS Ampliprep/COBAS Taqman HIV-1 Test (Roche Diagnostics Division, Basel, Switzerland).

### Isolation of Peripheral Blood Mononuclear cells

Peripheral blood mononuclear cells (PBMCs) were isolated within 3–5 hours of sample collection using Ficoll Hypaque in LeucoSep tubes. Cells were washed twice in Hanks Buffered Salt Solution with 0.1% gentamycin and counted using a capillary cytometer (Guava technologies, Hayward, CA). Cells (2×10^6^ cells/ml) were then rested overnight at 37°C in a 5% CO_2_ atmosphere in RPMI 1640 medium with GlutaMAX and 25mM HEPES (Gibco, Scotland) supplemented with 20% fetal bovine serum (Gemini Bio-Products, USA) and 0.1% gentamycin (R20).

### mRNA extraction and qRT-PCR

mRNA was extracted from one million PBMCs per sample using QIAamp RNA mini kit (QIAGEN, Germany). The mRNA was immediately converted to cDNA using the Applied Biosystems High-Capacity cDNA Archive Kit (Applied Biosystems, Foster City, CA) and the GeneAmp PCR System 9700 (Applied Biosystems, Foster City, CA). cDNA was frozen at −20°C until needed. Multiplexed real-time reverse transcriptase PCR was then performed using TaqMan Universal PCR Master Mix (Applied Biosystems, Foster City, CA.) on the 7500 Real Time PCR System (Applied Biosystems, Foster City, CA). *FOXP3* expression was assessed using a fluorescently labelled probe (TaqMan Gene expression assays, probe Hs00203958_m1, Applied Biosystems, Foster City, CA) relative to expression of *gapdh* (Pre-Developed TaqMan Assay Reagents Control Kit, Applied Biosystems Foster City, CA). All PCR reactions were run in triplicate in the presence of a blank control tube.

### Cell stimulations

PBMC were rested overnight in R20 medium at 2×10^6^ cells/ml with analysis of unstimulated samples on day 1. For assessment of cell proliferation, an equal volume of 10µM CFSE (Molecular Probes, Netherlands) was added to the unstimulated PBMC suspension. Samples were vortexed and incubated at room temperature in the dark for 7 minutes. To quench the CFSE reaction, a double volume of ice cold FBS was added. Samples were again vortexed and incubated at room temperature in the dark for 1 minute. PBMC were washed twice with warm RPMI 1640 medium with GlutaMAX and 25mM HEPES (Gibco, Scotland) supplemented with 10% human serum AB (Gemini Bio-Products, USA) and 0.1% gentamycin (R10) to remove excess CFSE. 2×10^6^ CFSE stained cells were seeded in a 24-well culture plate (Nunc, Denmark) in R10 medium to a final volume of 2ml. Stimulated samples were treated with 0.1 ug/ml stimulatory anti-CD3 mAb (12F6). Unstimulated samples were used as negative controls. Plates were incubated at 37°C, 5% CO_2_ for 4 days.

### Intracellular cytokine staining

Harvested PBMC (both those rested overnight and those cultured for 4 days) were stained for intracellular FOXP3 and CTLA-4 expression and surface expression of CD25, GITR, CD3 and CD4 using the FOXP3 staining set (eBiosciences, UK) according to manufacturer's instructions. Mean FOXP3% is reported from triplicate measurements. Preliminary experiments with propidium iodide showed efficacy of cell permeabilisation to be above 98%.

Antibody-fluorochrome conjugates used included CD3 APC, CD3 PerCP, CD4 FITC, CD4 PerCP, CD8 FITC,CD25 APC (BD Biosciences, San Jose, CA), GITR APC, CTLA-4 FITC (R&D Systems, Minneapolis, USA) and FOXP3 PE (clone PCH101, eBiosciences, UK).

### Flow cytometry

Flow cytometry was performed using FACSCalibur (BD Biosciences) and LSRII (BD Biosciences) flow cytometers with acquisition enabled by CellQuest Pro or FACSDiva software (BD Biosciences) respectively. Colour compensation was achieved using an appropriate single fluorochrome-labelled sample. Data was analysed using FlowJo 6.4.2 (TreeStar, USA). For CD25 quantitation, expression of CD25 on CD4^−^ T cells was used as a reproducible reference point.

50 000 to 1 million events were collected per sample. There was no use of biexponential axes. Not all samples were available for analysis of all parameters, depending on CD4 count and numbers of PBMCs available for culture.

### Statistical Analysis

Statistical analyses were performed using SPSS version 15 and GraphPad Prism version 4.0. Groups were compared by Mann Whitney analysis and correlations performed using the Spearman correlation coefficient. Significance was chosen at the 5% level. Group medians are reported.

## Results

### CD4 counts of HIV-infected subjects

HIV infected subjects had CD4 counts ranging from 0 to 712 cells/ul (median CD4 count 216 cells/ul) with 48% of patients <200 cells/ul group, 19% 200–350 cells/ul and 33% >350 cells/ul.

### HIV infected subjects display higher percentages of CD4^+^ T cells that express FOXP3 despite lower absolute numbers

FOXP3 expression in CD4^+^ T cells has been reported to confer a regulatory phenotype and may be dysregulated in HIV infection. To explore whether FOXP3 expression by CD4^+^ T cells differs in HIV infected individuals compared with controls, we isolated PBMCs and performed intracellular cytokine staining after overnight rest. In the control group, 3.72% of CD3^+^CD4^+^ T cells were positive for FOXP3 (range from 1.3–7.5%), in keeping with that described in the literature [10], [56]. The HIV infected group showed significantly elevated FOXP3 expression when expressed as a percentage of total CD3^+^CD4^+^ cells (median of 8.11%, range 1.33–26.27%, P = 0.002, Figure 1B,C). Absolute numbers of FOXP3^+^ CD4^+^ T cells were lower in HIV positive patients than controls (12.6 cells/µl versus 30.47 cells/µl, P = 0.005, Figure 1C) because of the lower CD4^+^ T cell count. Similarly, there was lower FOXP3 mRNA levels in HIV positive patients versus controls (0.92 vs 1.35, P = 0.03, data not shown).

FOXP3 expression in CD8^+^T cells, while found at a much lower proportion than CD4^+^ T cells, has also been reported [9], [52], [56], [57], [61], [62], [63], [64] and may confer regulatory functions. It is unknown if there is an alteration in CD8^+^ T cell FOXP3 expression during HIV infection. We found FOXP3 expression at low levels in the CD8^+^ T cell population but no significant difference between the control and HIV infected groups (0.20% of CD3^+^CD4^−^ versus 0.39% respectively). FOXP3 expression on the CD3^+^CD4^−^ population could be upregulated by antiCD3 stimulation (data not shown). There was however no significant difference in stimulated FOXP3 expression of CD4^+^ T cells or CD8^+^ (CD3^+^CD4^−^) T cells between the HIV and control groups.

### FOXP3 expression does not correlate with the activation marker CD25

**Figure 2:**
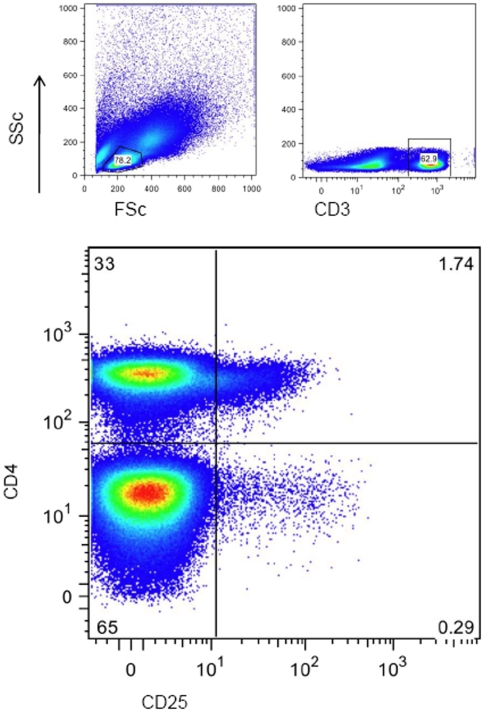
Representative plot illustrating CD25 gating strategy. Lymphocytes were gated according to forward and side scatter. CD3 positive lymphocytes were selected. CD25 expression on the x-axis was plotted against CD4 on the y-axis. A gate was set according to CD25 expression on the CD4^−^ population. This gave a percentage of CD3^+^CD4^+^CD25^+^ lymphocytes as a percentage of CD3^+^ (figure in top-right quadrant). This percentage was then used to calculate the percentage of CD3^+^CD4^+^CD25^+^ lymphocytes as a percentage of the CD3^+^CD4^+^ population (CD4 population being top-left and right quadrants added together).

CD25 is often used as a marker of regulatory T cells but can also act as an activation marker. CD25 expression in humans appears as a continuum of expression rather than a discrete positive and negative population, therefore to ensure reproducible gating, CD25 was gated by selecting the total CD3^+^ population and plotting CD25 against CD4. FOXP3 expression on the CD4^−^ population was used to set the positive gate. The resulting percentage obtained was used to calculate the CD25 expression as a percentage of the total CD3^+^CD4^+^ population (Figure 2).

CD25 expression gated in this manner did not differ significantly between the HIV infected and control group. While FOXP3^+^T helper cells were visually CD25^high^ as expected, FOXP3% showed no significant correlation with CD25 expression in either the control or HIV infected group. CD25 expression did not show a statistical correlation with either viral load or CD4 count.

Examining other postulated markers of regulatory T cells, CTLA4^+^FOXP3^+^ coexpression (as a percentage of CD3^+^CD4^+^ cells) was higher in the HIV group than the controls (0.78% versus 0.39%, P = 0.009, Figure 2A). There was no significant difference between the two groups in CD25^+^, CTLA-4^+^, GITR^+^ or GITR^+^FOXP3^+^ coexpression.

### CD4^+^ T cell FOXP3 expression is negatively correlated with CD4^+^ T cell count and positively correlated with viral load

**Figure 3:**
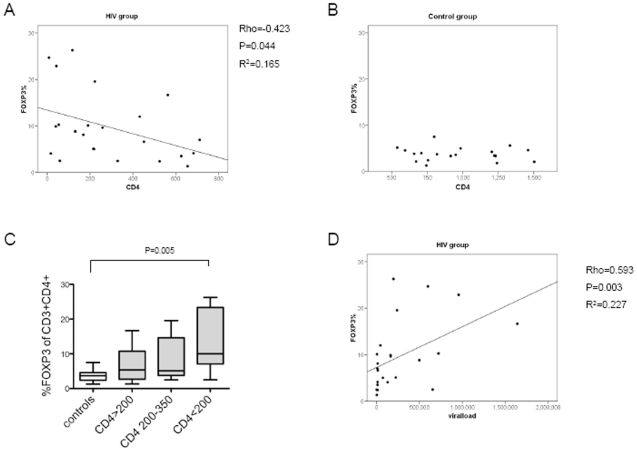
Correlation of FOXP3 with CD4 count and viral load. *Panel A*: CD4 count was negatively correlated with day 1 FOXP3 percentage in the HIV infected group. *Panel B*: The control group showed no correlation between CD4 count and FOXP3 percentage. Panel C: FOXP3 percentage stratified by CD4 count. *Panel D*: FOXP3 percentage was positively correlated with viral load.

As FOXP3 expression may be affected by the degree of immunodeficiency, we analysed the relationship between the CD4^+^T cell count and the percentage of CD4^+^T cells expressing FOXP3. There was a negative correlation demonstrable between CD4 count and FOXP3 percentage in the HIV infected group (rho = −0.423 P = 0.044, Figure 3A) but no significant correlation in the control group (Figure 3B). Stratification based on CD4 count revealed that it was only the samples with CD4 count <200 cells/µl that showed a marked elevation in FOXP3 percentage (median 10%), while there was no elevation in FOXP3 percentage in samples with higher CD4 counts (5.09% in samples with CD4 counts of 200–350 cells/µl and 5.36% in samples >350 cells/µ) (Figure 3C).

As anticipated, there was a significant negative correlation between viral load and CD4 count (rho = −0.538 P = 0.005, data not shown). We further determined that FOXP3 percentage correlated positively with viral load (rho = 0.593 P = 0.003, Figure 3D).

### High FOXP3 frequencies in HIV infected individuals are not due to increased FOXP3^+^ T cell lifespan nor increased T cell proliferation

In order to assess the mechanism by which the FOXP3 frequency of total CD4 T cells is elevated in HIV infected patients, we analysed FOXP3 expression in four-day cultures with and without stimulation through the T cell receptor with anti-CD3.

**Figure 4:**
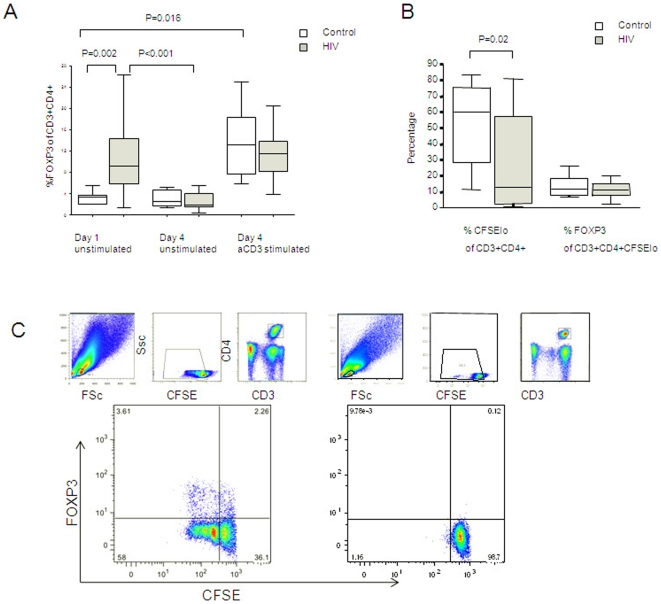
FOXP3 expression after T cell receptor stimulation. *Panel A*: FOXP3 expression as a percentage of T lymphocytes at baseline and after 4 days of cell culture, with and without T cell receptor stimulation with anti-CD3. *Panel B*: Proliferation of total CD4^+^ T cells and FOXP3^+^ expression in proliferated CD4^+^ T cells following T cell receptor stimulation with aCD3. *Panel C*: Representative plot of FOXP3^+^ expression in proliferated T cells (left-hand plot) following anti-CD3 stimulation compared with an unstimulated sample(right-hand plot). The small plots above show ancestry – lymphocytes were gated; followed by exclusion of events with high CFSE; followed by selection of CD3^+^CD4^+^ T cells. Proliferation is demonstrated by halving of CFSE fluorescence in cells that have divided (large plots below). In the anti-CD3 stimulated sample, FOXP3 expression is noted in cells which have proliferated (top-left quadrant) as well as those that have not proliferated (top-right quadrant).

Unexpectedly, unstimulated FOXP3 expression, expressed as a percentage of CD3^+^CD4^+^ cells, was significantly lower on day 4 than on day 1 in the HIV infected group (1.95% versus 9.75%, P<0.001) whilst there was no significant drop in expression in the control group (2.56% versus 3.36%) (Figure 4A). This suggests that FOXP3^+^ cells were not maintaining high levels due to a longer lifespan than FOXP3^−^ cells. After stimulation with anti-CD3, however, day 4 FOXP3 expression in the HIV infected group was comparable with baseline and with day 4 control group levels(Figure 4A), suggesting that mechanisms of FOXP3 upregulation after T cell receptor triggering are intact in HIV infected individuals.

To assess whether FOXP3 upregulation was due to increased CD4^+^T cell proliferation or increased expression in proliferated CD4^+^ T cells, we assessed FOXP3 expression following anti-CD3 stimulation of CFSE stained cells. Total CD4^+^ T cells proliferated less in the HIV infected group than in the control group (13.09% of CD3^+^CD4^+^ cells versus 60.08%, P = 0.02, Fig 4B). FOXP3 was upregulated in daughter cells (Figure 4C) but the percentage of proliferating cells expressing FOXP3 did not differ in the HIV infected and control groups (11.31% versus 11.78% Figure 4B).

Together, these findings suggest that high FOXP3 percentages in HIV infected patients is not due to longer lifespan, increased cell proliferation nor increased expression in proliferated cells. We conclude that the increased FOXP3 frequencies in HIV infected patients is likely due to upregulation of FOXP3 expression by individual CD4^+^ T cells, possibly following antigenic or other stimulation.

## Discussion

We used intracellular cytokine staining to analyse FOXP3 expression and other postulated markers of regulatory T cells (CD25, CTLA4 and GITR) in HIV infected individuals. We found that HIV infected individuals showed a significantly higher percentage of CD4^+^ T cells that expressed FOXP3 compared with control individuals. The percentage of FOXP3 expressing CD4^+^ T cells correlated negatively with CD4 count and positively with viral load. Due to their lower absolute CD4 count, the absolute number of FOXP3 expressing cells, as well as FOXP3 mRNA expression, was found to be lower in HIV infected individuals than in controls.

The finding of higher FOXP3 levels in patients with lower CD4 counts and higher viral loads is in keeping with the findings of others [11], [37], [65]. The inverse relationship between CD4 count and FOXP3 expression has not been consistently described and is well illustrated in our sample group possibly due to the inclusion of HIV infected patients with very low CD4 counts.This relationship also explains why studies with patients with relatively high CD4 counts at enrolment may have failed to demonstrate elevations of FOXP3 expression in peripheral blood. If the data is stratified into patients according to CD4 count, it is only the patients with CD4 counts below 200 cells/µl who show a marked elevation in FOXP3 percentage of CD3^+^CD4^+^ T cells (median 10%) while there is no significant difference in FOXP3 percentage between the 200–350 cells/µl or >350 cells/µl group and the control group (Figure 3C).

We noted that FOXP3 positivity was not limited to the CD4^+^ T cell subset but was also observed on other CD4^−^ T cells, albeit at much lower levels. Interestingly, FOXP3 upregulation after anti-CD3 stimulation, was noted on CD4^−^ T cells as well as on CD4^+^ T cells (data not shown). FOXP3 expression in CD8^+^ T cells has been described [9], [52], [56], [57], [61], [62], [63], [64], as have other subsets of FOXP3^−^ CD8^+^ cells with suppressor function [64], [66], [67], [68], [69]. In contrast to the CD4^+^FOXP3^+^ subset, we saw no significant difference in unstimulated or anti-CD3 stimulated percentages of CD4^−^FOXP3^+^ cells between the control and HIV infected groups.

HIV infected individuals are known to exhibit high levels of T cell activation and debate reigns as to whether FOXP3 is expressed by activated T cells that do not possess suppressive functions [52], [56], [58]. In this study, stimulated FOXP3 expression did correlate with *in vitro* proliferation of both CD4^+^ and CD8^+^ T cells to anti-CD3 stimulation but there was no correlation between expression of FOXP3 and the activation marker CD25. The correlation of FOXP3 with cell proliferation may suggest that FOXP3 acts as a marker of cell activation, however its discordance with CD25 expression indicates otherwise. The lack of statistical correlation between FOXP3 and CD25 expression, gated in a reproducible manner, lends weight to the argument that the elevated FOXP3 levels are not merely a marker of activated cells in HIV infection, in keeping with findings [70] describing dissociation between FOXP3 mRNA and CD25 expression in a SIV model. We took great care to gate CD25 using a reproducible gating strategy, namely using CD25 expression on CD4^−^ T cells as the cutoff for CD25 positivity. This proved a more reliable strategy than setting an arbitrary threshold for CD25^+^ and CD25^++^. Future studies should, however, include reference beads to allow more accurate CD25 determination. In previous studies, FOXP3 has also been shown not to correlate with the activation markers CD69 [31] or CD38 [9] although there was a correlation with HLA-DR expression in CD4^+^ T cells in the latter study.

CTLA-4 and GITR did not coincide with FOXP3 expression ie cells did not co-express FOXP3 with CTLA4 or GITR (Figure 1c). This is in keeping with findings by Weiss *et al.* [8] of intracellular CTLA-4 positivity in only 30% of Tregs (identified by CD4^+^CD25^hi^ expression) and Lim *et al.* [9] who found CTLA-4 or GITR expression in less than 10% of Tregs (CD4+CD25+CD127^lo^). A limitation of this study is that CD127 had not been included, which may have given additional discriminatory power of the Treg subset from activated cells. We therefore limited our functional analysis to FOXP3 expression.

The finding of increased percentages of CD4^+^ T cells expressing FOXP3 in HIV infected individuals suggests that an imbalance of regulatory to effector T cells could be responsible for susceptibility to opportunistic infection. Higher FOXP3 expression could be the result of one of four mechanisms: upregulated expression of FOXP3 in individual CD4^+^ T cells compared with HIV negative individuals, increased proliferation of cells expressing FOXP3^+^ cells, increased lifespan of FOXP3^+^ cells or increased cell death of FOXP3^−^ CD4^+^ T cells.

To analyse the mechanism of increased expression of FOXP3 amongst CD4^+^ T cells from HIV infected individuals, we cultured PBMCs from HIV infected and uninfected individuals in the presence of a stimulating antibody against the T cell receptor (anti-CD3). We found FOXP3 to be markedly upregulated by antigenic stimulation with anti-CD3 stimulation.

We did not directly address the lifespan or susceptibility to cell death of FOXP3 expressing cells in this study, however we noted that during unstimulated culture conditions, the elevated FOXP3 levels in the HIV infected groups declined rapidly to control levels. This is in keeping with a previous study suggesting that Tregs (CD3^+^CD25^hi^) are highly susceptible to apoptosis *in vitro* due to low levels of the antiapoptotic molecule Bcl-2 [54] and suggests that they do not have a longer lifespan than FOXP3^−^ cells.

In addition, FOXP3 expression in the HIV infected group could be rescued by anti-CD3 to levels comparable with and the stimulated control group. We explored whether this was due to increased CD4^+^ T cell proliferation or increased FOXP3 expression in cells which had proliferated. Using dual labelling with CFSE and FOXP3 we found that total CD4^+^ T cell proliferation was lower in the HIV group than in the control group, and the percentage of proliferated cells that expressed FOXP3 was similar in both groups. Thus the mechanism by which FOXP3 expression was upregulated *in vitro* in response to anti-CD3 stimulation is not likely to be due to CD4^+^ T cell proliferation. Together, these findings suggest that elevated FOXP3 percentages in HIV infected individuals is due to upregulation of FOXP3 expression in individual CD4^+^ T cells in HIV, likely due to antigenic or other stimulation. The downstream effect of FOXP3 upregulation was not directly addressed in this study. Transient FOXP3 upregulation after T cell receptor engagement may indicate that FOXP3 is an activation marker, however does not exclude the possible acquisition of suppressive potential after activation.

The debate over FOXP3 as activation marker versus marker of suppressive activity may eventually prove both sides right. The mechanisms of activation-induced cell death in healthy individuals is poorly understood, but every activated cell must eventually die or be suppressed, otherwise result in leukaemia. FOXP3 may indeed be both an “activation marker” and a marker of cells that have become unable to carryout effector functions.We have shown that FOXP3 expression was transient and could be lost and restimulated in HIV infected patients. Thus there did not seem to be an increase in number of natural Tregs with lineage-dependent FOXP3 expression in these patients, rather an increase in FOXP3 expression induced peripherally. This may be a result of cell activation, but the question remains as to why the increased numbers of activated T cells in HIV infected individuals fail to clear pathogens and are ineffectual in their actions. While not directly addressed by this study, it is plausible that continuous stimulation through the T cell receptor in HIV infected individuals may induce chronic rather than transient FOXP3 upregulation, resulting in dysfunctional effector T cells. A difference in function between FOXP3 and other activation markers may be an explanation for the discordance found in this study between FOXP3 expression and expression of the activation marker CD25.

It is recognised that CD4 count is not a perfect prognostic tool for monitoring of HIV infected individuals, with some patients appearing well even at low CD4 counts and some patients doing poorly even with relatively high counts (in our study one patient with a CD4 count of 2 appeared healthy). We suggest that FOXP3 expression in the CD4^+^ T cell subset may prove a more accurate prognostic tool to monitor disease progression and response to antiretroviral therapy. Longitudinal studies should be conducted to assess the use of FOXP3 frequency as a prognostic monitoring tool.

The inclusion in our HIV patient cohort of some HIV infected individuals with active Tuberculosis can be criticised, as there is evidence suggesting changes in Treg number or function result from Tuberculosis alone [71], [72], [73], [74], [75], [76], [77], [78], [79], [80], [81], [82], [83] although these studies remain inconclusive. Roberts *et al.* [84] found no difference in the levels of CD4^+^CD25^+^ Tregs in active tuberculosis cases compared with latently infected controls in unstimulated peripheral blood mononuclear cell cultures. Many of these studies, as with studies of Tregs in HIV infection, have assessed *FOXP3* mRNA expression rather than FOXP3 protein expression at the single cell level. Further, most quantitated Tregs based on CD25 expression without the use of a reference marker by which to set the CD25 gate. We conducted a subgroup analysis which showed no significant difference in levels of FOXP3 expression in HIV infected individuals with and without Tuberculosis (Figure 1A). Additionally, it can be hypothesized that any defect in Treg number or function may be of a similar nature in both HIV and Tuberculosis, given their propensity to occur simultaneously. Thus the co-infected group may illustrate more extreme changes not demonstrable in a small group of HIV infected patients alone.

In conclusion, we have shown that HIV infected individuals had significantly higher percentages of CD4^+^ T cells positive for FOXP3 than HIV uninfected individuals and that this is likely due to upregulation of FOXP3 expression by CD4^+^ T cells. FOXP3 expression as a percentage of CD4^+^ T cells correlated positively with viral load and negatively with CD4 count, with a marked elevation in FOXP3 percentage in patients with CD4 counts below 200 cells/µl. While correlation does not imply causation, this data support the hypothesis that FOXP3 plays a functional role in disease progression and may suppress responses to pathogens. The basis of the increased FOXP3 expression appears to be upregulation of FOXP3 expression by individual CD4^+^ T cells following T cell receptor stimulation. FOXP3 expression as a percentage of the CD4^+^ T cell population is a potential prognostic marker of disease progression.
